# Understanding and Addressing Older Adults’ Needs During COVID-19

**DOI:** 10.1093/geroni/igaa019

**Published:** 2020-06-02

**Authors:** Laura P Sands, Steven M Albert, J Jill Suitor

**Affiliations:** 1 Department of Human Development and Family Science and Center for Gerontology, Virginia Tech, Blacksburg; 2 Department of Behavioral and Community Health Sciences, University of Pittsburgh, Pennsylvania; 3 Department of Sociology and Center on Aging and the Life Course, Purdue University, West Lafayette, Indiana

Four months have passed since the World Health Organization (WHO) identified coronavirus disease 2019 (COVID-19) as a public health emergency, and 3 months since it declared the outbreak of COVID-19 a pandemic. WHO posted guidelines to reduce the spread of COVID-19, including isolating those with the virus, quarantining those exposed to the virus, and social distancing. These guidelines have been adopted across the globe, and there is evidence that following these guidelines slows transmission of the disease. Some have suggested that cases have reached a plateau, yet these conclusions are based on data drawn from reports of identified cases, which are not necessarily representative of all cases of COVID-19. This is problematic for designing guidelines and policies that are intended to protect the health of the entire population and the health of those who may be particularly vulnerable to COVID-19, including older adults. The pandemic has created difficulties for all, so we must be thoughtful about our pathway for returning to a life that is not threatened by COVID-19. Guidelines and policies for charting that pathway should be based on high-quality data, scientific knowledge, and ethical decision making.

## Older Adults’ Risk for Severe Disease and Mortality from COVID-19

Adults aged 65 and older are particularly vulnerable to severe disease and death due to COVID-19 (1,2), but the reasons for their vulnerability to COVID-19 are not understood. Some suggest that age-related increases in multimorbidity, declines in immunity, and increases in inflammatory pathways increase older adults’ vulnerability to COVID-19 (2,3). High-quality studies investigating mechanisms underlying older adults’ high rates of disease severity and mortality are urgently needed to inform evidence-based methods for preventing and treating COVID-19. Others suggest that older adults’ vulnerability is due, in part, to their higher likelihood of living in long-term care settings; in fact, nursing home patients account for half of COVID-19-related deaths among older adults (4,5). However, this pattern of increased risk varies considerably among such settings, suggesting that research is urgently needed to identify why some nursing homes were inadequately prepared to stem the spread of COVID-19 in their facilities. Others suggest that existing health disparities amplify older adults’ vulnerability to COVID-19. In the United States, hospital and mortality rates are two times higher for Black/African Americans and Hispanics than Whites (6). Black and Latino older adults are less likely to reside in long-term care settings than White non-Latinos; thus, in contrast to Whites, their higher rates of severe disease and mortality are both striking and unlikely due to living in long-term care settings. Research is needed to assess and address racial and ethnic disparities during the COVID-19 crisis (7). In addition, the risk of long-term morbidity among older adults who survive acute infection is also unclear. It is critical that scientists with expertise in aging tackle these complex issues with the goal of informing the development of interventions, whether they be focused on developing treatments for COVID-19, processes for providing care, or access to health care.

There are many challenges in conducting research among older adults during the pandemic. To date, nearly all health and mortality findings have relied on data from confirmed cases. Data from confirmed cases likely underestimate the population rate of COVID-19 and overestimate disease severity and mortality rates (8). In addition, reports of racial and ethnic disparities in COVID-19 are from reported cases; depending on the source of data, many cases are missing data for race and ethnicity (6,7). Carefully designed studies by experts in gerontology, geriatrics, and geroscience are needed to chart the pathway to moving toward a time when the lives of older adults are not threatened by COVID-19.

## Social Distancing and Older Adults’ Health and Well-being

Social distancing has been widely adopted as a tool for reducing the spread of COVID-19 (9). There is much to learn about older adults’ experiences with social distancing. For example, it is not known whether older adults who receive health and personal care in their homes are receiving sufficient care to maintain their health. Similar concerns have been voiced about the health consequences of closing Adult Day Centers, Cardiac Rehabilitation facilities, and other facilities that address the health and social needs of older adults. In addition, some suggest that social distancing and fear of the virus have led to reductions in older adults’ use of routine health care, access to food, and physical and social activities. Reduction in these activities may in turn increase morbidity among older adults (10). To inform guidelines and policy for COVID-19, research is needed to assess the impact of social distancing on older adults’ health, functioning, and well-being. Collecting data from older adults is particularly challenging during the COVID-19 crisis. Although web-based surveys seem to be an appealing alternative to in-person interviews, the few older adults who use web-based technology are not representative of older adults who are most vulnerable to social distancing (11). Researchers are encouraged to use recruitment and data collection strategies designed to collect representative samples of older adults with health, functional, and social barriers to participating in research (12).

## The Reframing Aging Initiative: Identifying Opportunities for Older Adults

The Reframing Aging Initiative, a collaboration of eight aging organizations including The Gerontological Society of America (13), reminds us that it is equally important to identify characteristics of older adults that increase their resilience to COVID-19 and potential consequences of social distancing. Identification of these characteristics will inform interventions, not just for older adults, but for others who face COVID-19-related challenges to their health, functioning, and well-being. In addition, the Reframing Aging Initiative reminds us of the value of opportunities to innovate existing systems to reduce poor health outcomes during and after the pandemic. For example, a recent article describes a new system of integrating home-based long-term care with primary care. Integration involves long-term care aides contacting primary care physicians when they discover changes in a care recipients’ health status (14). This is just one example of the potential impact of addressing COVID-19 challenges in health care. Similar to natural disasters, the pandemic has forced us to recognize how we fall short in ensuring that individuals’ basic health, social, and daily needs are met.

At the time of writing this editorial, there is very limited scientific data guiding our understanding of older adults’ vulnerability to COVID-19. The Editors of *Innovation in Aging* encourage submission of research articles on COVID-19 that have high potential for translating findings to effective guidelines and policies that reduce the threat of COVID-19 for vulnerable older adults. In light of the difficulties of initiating new research at this time, we have not set a deadline for submissions. Instead, we encourage researchers to conduct conceptually sound, methodologically rigorous research that will inform policies and guidelines for COVID-19 and future delivery of care.
